# Palliative management of painful pelvic bone metastasis using percutaneous thermal ablation and cement-augmented osteoplasty: A case report

**DOI:** 10.1016/j.ijscr.2025.111154

**Published:** 2025-03-14

**Authors:** Bangkit Primayudha, Muhammad Naseh Sajadi Budi Irawan, Rizwandha Noviar Azmi, Teguh Setiawanto

**Affiliations:** Orthopaedics and Traumatology Department, Hasan Sadikin Hospital, Padjajaran University, Bandung, Indonesia

**Keywords:** Metastatic bone disease, Breast cancer, Supracetabular lesion, Radiofrequency ablation, Cementoplasty, Minimally invasive surgery, Pain management

## Abstract

**Introduction and importance:**

Bone metastases are common in advanced malignancies, particularly from breast and prostate cancers, leading to significant morbidity due to pain, functional impairment, and skeletal-related events. This case report discusses a multidisciplinary approach using minimally invasive techniques for managing a challenging supracetabular metastatic lesion.

**Case presentation:**

A 46-year-old female with metastatic bone disease in the right supracetabular pelvic region secondary to primary breast cancer presented with severe pain (Numeric Analog Scale [NAS] 7–8) and functional impairment. Systemic bisphosphonate therapy with zoledronic acid was initiated. The patient underwent a combination of percutaneous radiofrequency ablation (RFA), osteoplasty, and cementoplasty under C-arm guidance. Medium-viscosity acrylic cement was injected to stabilize the lesion post-ablation.

**Clinical discussion:**

The procedure was successful, with significant pain relief (NAS reduced to 3–4) and improved functional stability. The patient experienced rapid recovery with minimal complications and reduced analgesic requirement. This case highlights the efficacy of combining RFA and cementoplasty for localized management of osteolytic metastatic lesions. RFA provided cytotoxic tumor ablation, while cementoplasty reinforced bone structure and contributed to additional pain relief. The outcomes align with existing literature demonstrating the benefits of these techniques in reducing pain and improving quality of life.

**Conclusion:**

Minimally invasive interventions such as RFA and cementoplasty are effective in managing symptomatic bone metastases, offering a viable alternative to traditional surgical methods. This approach underscores the importance of integrating systemic and localized therapies in advanced cancer care.

## Highlights

• Bone metastases are common, leading to significant modbidity due to pain, functional impairment, and skeletal-related events
• Percutaneous thermal ablation is a technique to induce tumor necrosis
• Cement-augmented osteoplasty is a procedure to stabilize bone and restore mechanical strength
• Multidisciplinary approach in managing metastatic bone disease with bisphosphonates, percutaneus thermal ablation, and cement-augmented osteoplasty provides comprehensive strategy with promising outcome

## 1 Introduction

Bone metastases occur in >1.5 million patients with cancer worldwide. They are frequent complications of many cancers but are especially common from tumors arising in the breast and prostate. Weakened bones due to skeletal metastases can lead to occurrence of skeletal-related events, such as fractures, spinal cord compression, bone pain, and disability, contributing substantially to both morbidity and mortality in patients with advanced cancer. In adults, the bone mass is maintained by continuous shaping and reshaping of the overall bone structure through a process called bone remodeling, which is a balance between the resorption of mineralized bone by boneresorbing cells (osteoclasts) and formation of new bone by bone-forming cells (osteoblasts) [1].

Bone remodeling is tightly regulated by systemic and local factors to maintain this balance at its physiological steady state [1]. Bone is one of the most common sites for metastasis in cancer. Much of the work performed to describe the natural history of bone metastases is based on autopsy studies and large case series from single institutions conducted several decades ago. Although bone is a frequent location for metastases from many malignancies, there are specific types of cancers that have a predilection for metastasis to the skeleton. Bone metastases in the pelvis significantly impair the patient's quality of life and require treatment. Surgery may be needed only if the patient has a good prognosis for solitary and late metastases at these sites. In such cases, wide resection or aggressive curettage may improve patient survival [1].

The ilium wing and the anterior pelvic arch may cause less dysfunction after en bloc resection and may not require reconstruction other than reinforcement with synthetic mesh to avoid visceral hernias. Periacetabular lesions are usually painful with weight bearing and are at risk of mechanical failure, resulting in progressive protrusio acetabuli. Therefore, surgical treatment and postoperative radiotherapy may be indicated to reduce pain, restore function, and allow early weight bearing. Cotten et al. used the vertebroplasty technique to treat osteolytic metastatic lesions around the acetabulum. Acetabuloplasty consisted of percutaneously injecting low viscosity acrylic cement into the osteolytic cavity. The main goal is to increase the resistance of bone metastatic lesions to compressive stress and reduce the risk of fractures. In addition, the exothermic reaction during cement polymerization may cause a local cytotoxic reaction to the tumor. Acetabuloplasty provided complete pain relief in 59 % of patients. Combining ablation treatment with cementoplasty may increase overall efficacy. In general, percutaneous treatment has a very low incidence of complications. The indications for acetabuloplasty are pain and impending fractures [2].

We present a case of metastatic bone disease involving the right supracetabular pelvis secondary to primary breast cancer. The patient received bisphosphonate therapy with zoledronic acid 4 mg administered biweekly. She subsequently underwent a minimally invasive surgical procedure comprising percutaneous radiofrequency ablation, osteoplasty, and cementoplasty. Postoperatively, the patient experienced a marked reduction in pain, with the Numeric Analog Scale (NAS) score decreasing from 7 to 8 preoperatively to 3–4. This case has been documented in accordance with the SCARE guidelines [3].

## 2 Case report

**Fig. 1:**
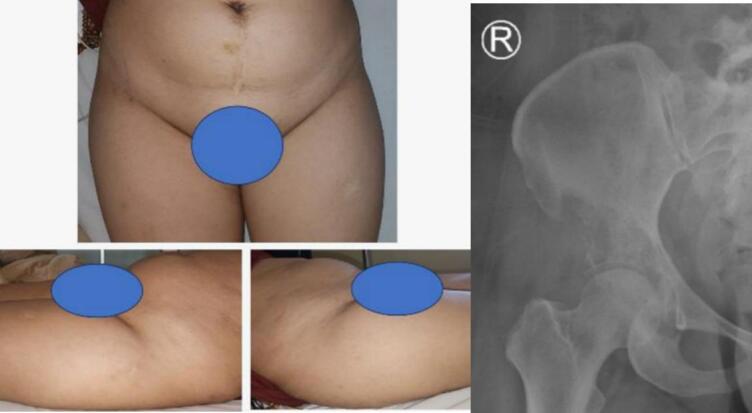
Clinical appearance at pelvic region (left). Pre-surgical radiographs (right).

A 46-year-old female patient presented to the outpatient clinic with excruciating pain in her right hip region and weakness in her right lower extremity. She had been wheelchair-bound due to these symptoms, which had persisted for two months prior the admission (Karnofsky score 50). She was a stage 4 breast cancer patient with metastases spread to the pelvis and lungs. The patient previously received systemic treatment with continuous bisphosphonate with zoledronic acid and palliative chemotherapy. A core biopsy confirmed the diagnosis of supracetabular metastatic carcinoma. She was subsequently hospitalized for pain management and prepared for a surgical intervention to address her condition. Informed consent for the procedure was obtained from the patient (Fig. 1).

Further examination of the pelvic region revealed no visible abnormalities such as lumps, scars, wounds, swelling, or discoloration. However, tenderness was noted over the right pelvic region, particularly in the area of the greater trochanter, accompanied by warmth but no crepitus. The patient was unable to complete the assessment of hip active and passive range of motion due to significant discomfort.

**Fig. 2:**
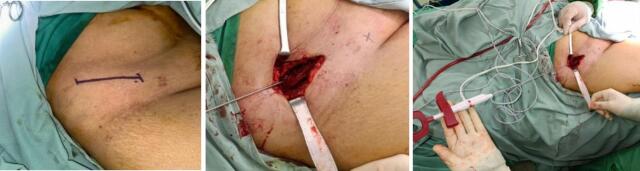
Design incision at right pelvic region (left). Pre-ablation with RF-Trochard ablation accesability (middle). Ablation at right supraacetabular region (right).

Radiographic evaluation, including an anteroposterior view of the pelvis, demonstrated a single permeative osteolytic lesion in the right supraacetabular region. To address this condition, we proceeded with surgical management using a percutaneous ablation technique. Under C-arm fluoroscopic guidance, we accessed the supraacetabular region with a 3.5 mm drill bit to create an open canal for the port d'entrée. Radiofrequency ablation (RFA) was then performed using the Radio-frequency (RF) Wolf system to thermally ablate the lesion (Fig. 2).

**Fig. 3:**
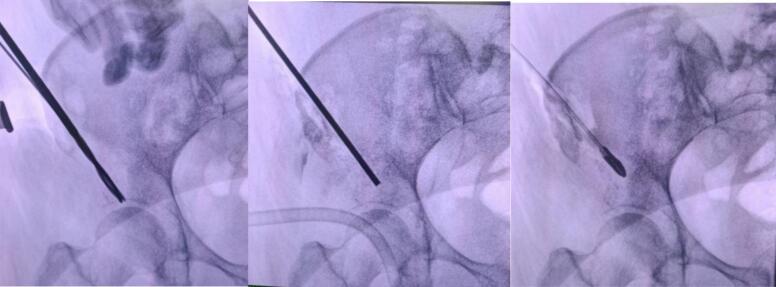
Underguided C-Arm intra operative. Open canal access (left). Ablation with RF-Trochard (middle). Cementoplasty balloon insertion (vertebroplasty instrumentation) at supraacetabular region (right).

We created an access hole using a drill and positioned the ablation trocar under C-arm fluoroscopic guidance. The RFA was performed intermittently using cooled 17-G length-adjustable electrode and a 200-W RF generator, over 10 min, with each cycle consisting of 20 s of continuous ablation followed by a 10-s rest interval. Upon successful completion of the ablation, we proceeded with osteoplasty and cementoplasty. The trocar was used to introduce cement into the superior acetabular region, ensuring proper fusion and stabilization of the affected area (Fig. 3).

**Fig. 4:**
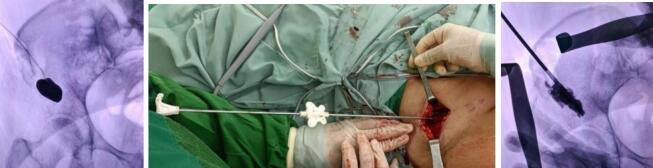
Underguided C-Arm intra operative. Inflated cement vertebroplasty balloon (left). Cement injection (middle). Cement filled at supraacetabular (right).

We positioned the vertebroplasty trocar balloon into the right supraacetabular region and carefully inflated it to create space within the lesion. Once the balloon was successfully inflated, we replaced it with the cement trocar to access the supraacetabular canal. Medium-viscosity cement was prepared 2 min after blending to avoid excessively sticky cement and injected into the supraacetabular region, with a total volume of 50 cc administered to stabilize the area effectively (Fig. 4).

**Fig. 5:**
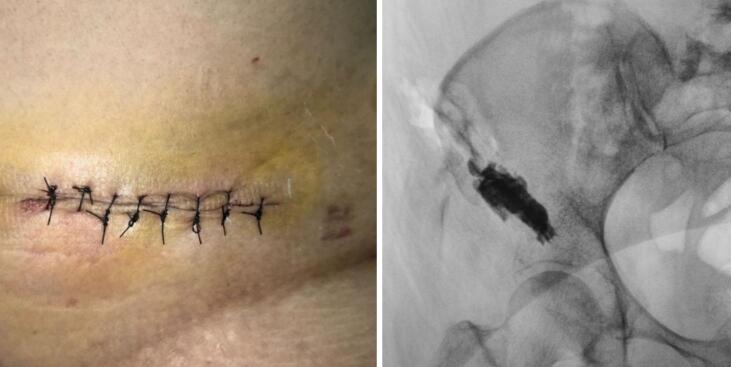
Post operative wound care with 5 cm incision (left). Radiograph post ablation, osteoplasty, and cementoplasty (Right).

On postoperative day 3, the patient was followed up without the need for analgesic substitution from the preoperative regimen. The patient reported a significant reduction in pain, with NAS score of 3–4. The patient was followed up at 3 and 6 month post operation to monitor the progress of pain management. Plain radiograph was used for evaluation (Fig. 5).

## 3 Discussion

Metastatic bone disease is a prevalent complication in patients with advanced malignancies, especially those originating from the breast and prostate. The patient presented in this case underscores the profound impact of metastatic bone lesions on quality of life, including pain, functional impairment, and the heightened risk of skeletal-related events. Treatment decisions for metastatic bone disease should focus on achieving pain relief, ensuring structural stabilization, and enhancing functional mobility [1,4].

The location of the metastatic lesion in the right supracetabular region posed unique challenges in terms of both symptomatology and treatment. A multidisciplinary team should be formed to discuss the needs of the patient before undergoing surgery, from the oncology department discussing further treatment options to the postoperative rehabilitation team discussing strength and mobility recovery if surgery is indicated. Supracetabular lesions are particularly debilitating as they affect the weight-bearing pelvic structure, leading to significant pain and risk of mechanical failure, such as pathological fractures or acetabular protrusion. Traditional management strategies, including systemic therapies like bisphosphonates and radiotherapy, may not sufficiently address localized pain or structural instability, therefore surgery is indicated [1,2].

The combined use RFA, osteoplasty, and cementoplasty in this case provided a minimally invasive yet effective solution to achieve the desired outcomes.

RFA is a thermal ablation technique that uses high-frequency alternating currents to generate heat, inducing tumor necrosis. Radiofrequency ablation operates like a typical electrosurgical device, with current flowing through electrodes to the body and exiting through a grounding pad. The size of the lesion created depends on factors such as tissue type, current duration, and temperature, with apoptosis occurring at temperatures between 46 °C and 60 °C, while temperatures above 100 °C can cause unnecessary tissue damage and reduce the effectiveness of treatment. In this case, RFA was critical for reducing tumor burden and achieving localized cytotoxicity. The use of RF Wolf radiofrequency ablation under C-arm guidance ensured precise targeting of the metastatic lesion, minimizing damage to surrounding tissues [2,5,6].

Duration of this procedure also counts, as studies concluded that little to no change was achieved in the extent of the ablation zone after approximately 3 min but that nevertheless, in clinical practice, the patients seemed to experience less recurrences when the duration of the RFA was increased to 6 min. Other studies even claimed to notice a difference in the clinical outcomes by increasing the ablation time to up to 15 min [7].

Osteoplasty and Cementoplasty: Following ablation, osteoplasty and cementoplasty were performed to stabilize the bone and restore its mechanical strength. The injection of medium-viscosity acrylic cement served two purposes [6]: (1) Structural support: It reinforced the weakened bone, reducing the risk of pathological fractures and improving weight-bearing capacity. (2) Pain relief: The exothermic polymerization of the cement likely contributed to additional cytotoxic effects on residual tumor cells, further alleviating pain. Intraoperative prevention of complications surrounding bone cement injection, such as bone cement leakage, should also be monitored. Currently, intraoperative C-arm fluoroscopy is still the most predominant approach for monitoring, yet its image quality is even inferior to that of plain radiographic images [9].

Clinical Outcomes and Benefits: The patient experienced significant pain relief, with the NAS score decreasing from 7 to 8 preoperatively to 3–4 postoperatively. This marked improvement highlights the efficacy of combining RFA with cementoplasty in managing localized metastatic bone lesions. Furthermore, the minimally invasive nature of the procedure facilitated rapid recovery, as evidenced by the patient's reduced analgesic requirements and early mobilization postoperatively [10]. With the increasing use of RFA in major cities and medical centers in Indonesia, this research can improve the efficacy and explore a better surgical approach.

Comparison with Existing Literature: The outcomes of this case align with findings from previous studies that demonstrate the efficacy of cementoplasty in reducing pain and improving structural integrity in osteolytic lesions. For example, Cotten et al. reported complete pain relief in 59 % of patients undergoing acetabuloplasty, with low complication rates. The integration of RFA further enhances this approach by directly targeting tumor tissue and addressing tumor-related pain [11].

Implications for Clinical Practice: This case underscores the importance of a multidisciplinary approach in managing metastatic bone disease from diagnostic to postoperative rehabilitation. The combination of systemic therapies like bisphosphonates and localized interventions such as RFA and cementoplasty provides a comprehensive strategy to address both the systemic and local effects of skeletal metastases. Additionally, minimally invasive techniques offer significant advantages over traditional surgical approaches, including reduced operative morbidity, shorter hospital stays, and quicker recovery [12].

## 4 Limitations and considerations

While the outcomes in this case were favorable, there are limitations to this approach: (1) Patient Selection: Not all patients with bone metastases are candidates for minimally invasive procedures. The prognosis, overall disease burden, and location of metastases must be carefully evaluated. (2) Long-term Outcomes: The long-term durability of pain relief and structural stability provided by cementoplasty in the context of progressive metastatic disease remains uncertain and warrants further study. (3) Complications: Although rare, complications such as cement leakage or thermal injury to adjacent structures may occur and should be monitored closely [12].

## 5 Conclusion

The successful management of a supracetabular metastatic lesion using a combination of RFA, osteoplasty, and cementoplasty demonstrates the efficacy of this minimally invasive approach in alleviating pain, improving structural integrity, and enhancing quality of life in patients with metastatic bone disease. This case adds to the growing body of evidence supporting the role of localized interventions as a valuable adjunct to systemic therapy in advanced cancer care.

## CRediT authorship contribution statement

Bangkit Primayudha as surgeon, study concept and design
Muhamad Naseh Sajadi Budi Irawan as surgeon, data analysis
Rizwandha Noviar Azmi as surgeon, writing paper
Teguh Setiawanto as surgeon, writing paper.

## Consent

Written consent was obtained from the patient for publication of this case report and accompanying images. A copy of the written consent is available for review by the Editor-in-Chief of this journal on request.

## Ethical approval

We certify this kind of manuscript does not require ethical approval (exemption) by the Ethical Committee of our institution at Hasan Sadikin Hospital, Bandung, Indonesia.

## Guarantor

Bangkit Primayudha.

## Funding

This research did not receive any specific grant from funding agencies in the public, commercial, or not-for-profit sectors.

## Declaration of competing interest

All authors disclose any conflicts of interest.
